# A Reinforcement-Learning-Driven Multi-Strategy Spherical-Vector Grey Wolf Optimizer for UAV 3D Path Planning

**DOI:** 10.3390/biomimetics11070470

**Published:** 2026-07-05

**Authors:** Anna Li, Yanqiang Yang

**Affiliations:** School of Mathematics and Science, Hebei GEO University, Shijiazhuang 050031, China; 240907010001013@hgu.edu.cn

**Keywords:** UAV, 3D path planning, reinforcement learning, grey wolf optimizer, spherical vector, strategy selection

## Abstract

Unmanned aerial vehicles (UAVs) have been widely used in surveying and mapping, inspection, emergency rescue, and environmental monitoring. However, effective path planning remains a key challenge in complex three-dimensional terrain, where UAVs must simultaneously cope with terrain undulations, no-fly zones, safety-clearance requirements, and trajectory-smoothness constraints. In addition, conventional intelligent optimization algorithms often suffer from search instability and premature convergence. To address these challenges, this study proposes a reinforcement-learning-driven multi-strategy spherical-vector grey wolf optimizer, termed TLQ-SGWO, where TLQ denotes the combined use of Tent–Logistic hybrid initialization and Q-learning search-strategy scheduling. In the proposed method, candidate trajectories are encoded using spherical-vector increments; Tent–Logistic hybrid initialization is introduced to enhance population diversity; and Q-learning is incorporated to adaptively select search strategies, thereby dynamically balancing exploration and exploitation. A comprehensive cost function integrating path length, threat avoidance, terrain clearance, and trajectory smoothness is further constructed to improve the feasibility and safety of the planned trajectories. Experiments are conducted on the CEC2017 benchmark functions and artificially generated complex mountainous terrain scenarios. On the CEC2017 benchmark suite, TLQ-SGWO achieves the best average rankings in both mean error and standard deviation among the seven compared algorithms, indicating a stronger balance between optimization accuracy and robustness. In artificial mountainous scenarios, TLQ-SGWO obtains the lowest mean path cost in three of the four scenarios and remains statistically comparable to the strongest hybrid baseline in the remaining scenario, while maintaining stable feasible 3D trajectories under increasing no-fly-zone complexity.

## 1. Introduction

Unmanned aerial vehicles (UAVs) are increasingly used in mapping, inspection, emergency rescue, environmental monitoring, and other autonomous missions. Advances in multi-sensor fusion and attitude control have improved autonomous inspection accuracy and have also raised the requirement for safe and reliable flight operation [1]. For autonomous UAVs, path planning aims to generate a safe, flyable, and low-cost trajectory between a prescribed start point and goal point. Existing surveys indicate that UAV path planning in complex environments must simultaneously consider mission constraints, environmental threats, computational requirements, and multi-objective trade-offs [2,3]. Compared with two-dimensional planning, 3D path planning in mountainous terrain further involves terrain undulation, no-fly zones, terrain clearance, maximum flight altitude, turning-angle constraints, and climb/descent smoothness. The resulting problem is therefore high-dimensional, nonlinear, and strongly constrained. In tasks such as 3D structural coverage, path planning must also consider coverage efficiency, structural geometry, and flight reachability [4].

Traditional path-planning methods include Dijkstra, A*, RRT, artificial potential fields, and grid search [5,6]. These methods are interpretable on structured maps, but search-space dimensionality, path-smoothness requirements, and susceptibility to local minima often constrain their performance in continuous 3D terrain. In addition to graph-search methods, LSTM-based real-time path planning has been investigated to improve UAV prediction and response under time-varying environmental information [7]. Swarm intelligence algorithms, including particle swarm optimization (PSO), grey wolf optimizer (GWO), whale optimization algorithm (WOA), and differential evolution, do not require gradient information and are suitable for complex non-convex optimization problems [8,9,10]. However, their performance may still be limited by unstable random initialization, fixed search-stage transitions, and insufficient robustness in complex threat regions.

Genetic algorithms (GAs) provide a complementary evolutionary-search paradigm for vehicle maneuverability and UAV path planning because crossover and mutation can preserve diverse candidate routes while recombining promising trajectory segments. Akopov et al. developed a parallel biobjective real-coded GA to improve maneuverability in a multiagent fuzzy transportation system, demonstrating the suitability of real-valued evolutionary search for balancing conflicting motion objectives [11]. Pehlivanoglu and Pehlivanoglu proposed an enhanced GA for autonomous UAV target-coverage path planning and showed that tailored genetic operators can improve route quality under task-specific constraints [12]. These studies highlight the relevance of GA-based search to continuous trajectory optimization and motivate its consideration when evaluating population-based UAV path-planning methods.

Hybrid evolutionary and swarm-intelligence algorithms have also attracted increasing attention because they can combine the population-diversity preservation of evolutionary operators with the fast information-sharing ability of swarm search. However, exhaustively comparing all hybrid variants is difficult because different hybrid algorithms often use different encoding schemes, operators, parameter settings, and computational budgets. Therefore, this study introduces RCGA-PSO as a representative hybrid continuous-optimization baseline, together with GA and PSO, to examine whether the proposed TLQ-SGWO remains competitive against both classical and hybrid population-based search mechanisms.

Spherical-vector path encoding describes a 3D UAV trajectory using radial step length, pitch angle, and horizontal heading angle, making the decision variables more consistent with the geometric characteristics of UAV motion. Previous studies have combined spherical vectors with PSO, angle-coded swarm intelligence methods, and improved swarm optimization algorithms, demonstrating the applicability of this encoding strategy to 3D UAV path planning [13,14,15,16]. In parallel, nonlinear convergence factors, event-triggered mechanisms, and adaptive weights have been introduced into GWO to improve 3D trajectory-planning performance [17].

Nevertheless, three issues remain insufficiently addressed. First, randomly initialized populations may concentrate in high-penalty terrain or threat regions, reducing early-stage search quality. Second, fixed GWO search strategies are difficult to adapt to different evolutionary states. Third, under dense no-fly-zone constraints, the population may stagnate around low-quality feasible corridors or obstacle boundaries. Studies on improved GWO and multi-strategy path planning have shown that adaptive strategies can alleviate exploration–exploitation imbalance and premature convergence [18,19,20,21]. Motivated by these observations, this study proposes a Tent–Logistic- and Q-learning-based multi-strategy spherical-vector grey wolf optimizer, abbreviated as TLQ-SGWO, for UAV 3D path planning. Here, TLQ refers to the combination of Tent–Logistic hybrid initialization and Q-learning search-strategy scheduling; rather than a separate variant of Q-learning. The hybrid initialization is designed to combine the nonlinear state dispersion of the Logistic map with the piecewise expansion of the Tent map, thereby reducing dependence on a single chaotic sequence. Within the spherical-vector SGWO framework, the proposed algorithm schedules search actions through Q-learning and combines hybrid initialization, controlled Lévy perturbation, Alpha local refinement, and a safety-oriented elite branch to improve search quality under complex 3D constraints.

The main contributions of this study are summarized as follows:

1. A spherical-vector multi-constraint path-planning model is constructed for complex 3D terrain. The model represents trajectory segments using spherical-vector increments and integrates path length, cylindrical no-fly-zone threat, terrain collision and clearance, trajectory smoothness, low-terrain guidance, and altitude constraints into a seven-term comprehensive cost function, thereby providing an optimization space consistent with UAV motion characteristics.
2. A Q-learning-based search-strategy scheduling mechanism is developed for SGWO. To balance exploration, exploitation, and stagnation handling under complex constraints, the search state is defined by the improvement level, population diversity, and iteration stage. The algorithm then adaptively selects local exploitation, standard search, enhanced exploration, and Lévy escape actions. In addition, hybrid initialization and heuristic seed paths are incorporated as an initial-distribution improvement module to provide higher-quality feasible search directions early in the search.
3. Controlled stagnation escape, Alpha local refinement, and safety-redundancy mechanisms are designed, and the algorithm is evaluated through a two-level experimental framework. CEC2017 benchmark testing is first used to examine the general continuous optimization capability. Artificial complex mountainous scenarios are then used for task-specific UAV path-planning validation, supported by algorithm comparisons, convergence analysis, ablation studies, and nonparametric statistical tests.

## 2. UAV 3D Path Planning Model

### 2.1. 3D Terrain and No-Fly Zone Model

This study considers a discrete 3D terrain region with the horizontal coordinate range $\left[1,N_{x}\right]\times \left[1,N_{y}\right]$, where the terrain elevation is given by the height matrix H(x,y). The UAV flies from the start point

(1) $$S=(x_{s},y_{s},z_{s}).$$

to the goal point

(2) $$G=(x_{g},y_{g},z_{g}).$$

The experimental scenarios are generated from artificial complex mountainous terrain, including ridges, valleys, and several cylindrical no-fly zones. Scenario 1–Scenario 4 are configured with 2, 3, 4, and 5 cylindrical no-fly zones, respectively, to investigate the influence of threat-density variation on algorithmic performance. Each no-fly zone $NF_{i}$ is represented by its center $\left(x_{i},y_{i},z_{i}\right)$, horizontal radii $\left(a_{i},b_{i}\right)$, half-height $c_{i}$, and threat intensity $\lambda_{i}$. For a path sampling point p=(x,y,z), the normalized horizontal distance is defined as

(3) $$d_{i}^{xy}\left(p\right)={\left(\frac{x-x_{i}}{a_{i}}\right)}^{2}+{\left(\frac{y-y_{i}}{b_{i}}\right)}^{2},$$

When $d_{i}^{xy}\leq 1$ and $z\in [z_{i}-c_{i},z_{i}+c_{i}]$, the sampling point lies inside the cylindrical no-fly zone and receives a high-intensity penalty. In the experiments, the cylindrical no-fly zones satisfy $a_{i}=b_{i}=r_{i}$.

### 2.2. Spherical-Vector Path Encoding

Assume that a candidate path contains *n* intermediate trajectory segments. The *k*-th trajectory segment is represented by the spherical vector

(4) $$\omega_{k}=\left(\rho_{k},\varphi_{k},\theta_{k}\right),\;k=1,2,\ldots ,n,$$

where $\rho_{k}$ is the radial step length of the *k*-th trajectory segment, $\varphi_{k}$ is the pitch angle, and $\theta_{k}$ is the horizontal heading angle. To make the search direction consistent with the overall direction from the start point to the goal point, the variable ranges are set as

(5) $$\rho_{k}\in \left[0,\rho_{\max}\right],\;\varphi_{k}\in \left[-\pi /4,\pi /4\right],\;\theta_{k}\in \left[\theta_{0}-\pi /4,\theta_{0}+\pi /4\right],$$

where

(6) $$\rho_{\max}=\frac{2∥G-S∥}{n},\;\theta_{0}=\mathrm{atan}2\left(y_{g}-y_{s},x_{g}-x_{s}\right).$$

The incremental mapping from the spherical vector to Cartesian coordinates is given by

(7) $$\left\{\begin{array}{c} x_{k}=x_{k-1}+\rho_{k}\cos\varphi_{k}\cos\theta_{k},\\ y_{k}=y_{k-1}+\rho_{k}\cos\varphi_{k}\sin\theta_{k},\\ z_{k}=z_{k-1}+\rho_{k}\sin\varphi_{k}. \end{array}\right.$$

During path generation, $x_{k}$ and $y_{k}$ are constrained within the valid map boundaries, and $z_{k}$ is corrected to be no lower than the terrain elevation at the current position plus a safety margin. This repair operation prevents candidate paths from becoming directly invalid during generation because of boundary violation or terrain penetration.

### 2.3. Comprehensive Cost Function

The path planning problem is transformed into a comprehensive cost minimization problem:

(8) $$\min_{\Omega }J\left(\Omega \right)=\sum_{i=1}^{7}b_{i}J_{i}\left(\Omega \right),$$

where $\Omega =\{\omega_{1},\ldots ,\omega_{n}\}$ denotes a candidate spherical-vector path. The complete trajectory nodes are defined as $p_{0}=S$ and $p_{n+1}=G$, and the intermediate nodes are generated by Equation (7). To detect the relationship between each trajectory segment and the terrain or no-fly zones, the *k*-th trajectory segment is uniformly sampled as

(9) $$q_{k,l}=p_{k}+\frac{l}{N_{s}-1}\left(p_{k+1}-p_{k}\right),\;k=0,1,\ldots ,n,\;l=0,1,\ldots ,N_{s}-1,$$

where $N_{s}=20$, namely each trajectory segment contains 20 equally spaced sampling points including the two endpoints. Let H(x,y) be the terrain-elevation query function. The terrain clearance of a sampling point is

(10) $$\delta_{k,l}=z\left(q_{k,l}\right)-H\left(x\left(q_{k,l}\right),y\left(q_{k,l}\right)\right).$$

The seven cost terms are summarized in Table 1 and defined as follows.

#### 2.3.1. 3D Path Length Function

The 3D path length measures the total distance traveled by the UAV along the planned trajectory from the start point to the goal point and is an important metric for evaluating trajectory economy. The planned path consists of the start point, *n* intermediate trajectory points, and the goal point, and is expressed as $P=\{p_{0},p_{1},\cdots ,p_{n},p_{n+1}\}$,

(11) $$J_{1}=\sum_{k=0}^{n}{∥p_{k+1}-p_{k}∥}_{2}=\sum_{k=0}^{n}\sqrt{{\left(x_{k+1}-x_{k}\right)}^{2}+{\left(y_{k+1}-y_{k}\right)}^{2}+{\left(z_{k+1}-z_{k}\right)}^{2}}.$$

#### 2.3.2. No-Fly Zone Threat Function

The no-fly-zone threat term $J_{2}$ is calculated according to the horizontal elliptical distance and altitude range of the cylindrical no-fly zones. For the *i*-th cylindrical no-fly zone, its center is $\left(x_{i},y_{i},z_{i}\right)$, its horizontal semi-axes are $a_{i},b_{i}$, its half-height is $c_{i}$, and its threat intensity is $\lambda_{i}$. The normalized horizontal distance from a sampling point q to the no-fly zone is

(12) $$d_{i}^{xy}\left(q\right)={\left(\frac{x\left(q\right)-x_{i}}{a_{i}}\right)}^{2}+{\left(\frac{y\left(q\right)-y_{i}}{b_{i}}\right)}^{2},$$

The vertical boundaries are $z_{i}^{-}=z_{i}-c_{i}$ and $z_{i}^{+}=z_{i}+c_{i}$, and the distance to the vertical boundary is

(13) $$\Delta z_{i}\left(q\right)=\min\left(|z\left(q\right)-z_{i}^{-}|,\;|z\left(q\right)-z_{i}^{+}|\right).$$

**Figure 1:**
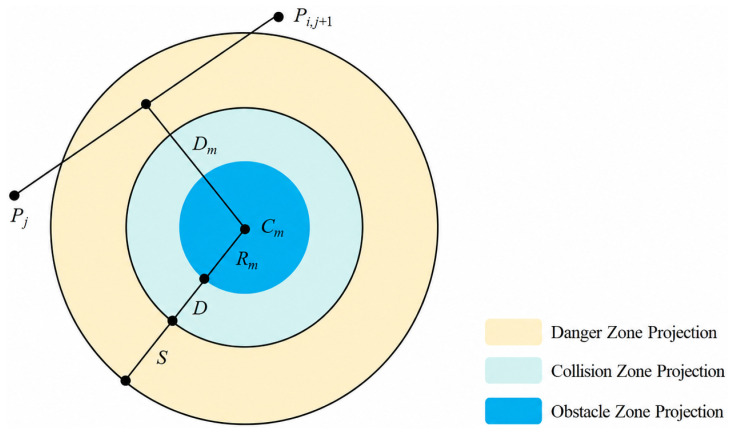
Schematic diagram of flight threat cost analysis.

The cylindrical threat penalty of a single sampling point is written as

(14) $$\Gamma_{i}\left(q\right)=\left\{\begin{array}{cc} M\lambda_{i}\left(3-d_{i}^{xy}\left(q\right)\right), & d_{i}^{xy}\left(q\right)\leq 1,\;z_{i}^{-}\leq z\left(q\right)\leq z_{i}^{+},\\ 180\lambda_{i}{\left(35-\Delta z_{i}\left(q\right)\right)}^{2}, & d_{i}^{xy}\left(q\right)\leq 1,\;\Delta z_{i}\left(q\right)<35,\\ 14000\lambda_{i}{\left(2.25-d_{i}^{xy}\left(q\right)\right)}^{2}, & 1<d_{i}^{xy}\left(q\right)\leq 2.25,\;z_{i}^{-}-35\leq z\left(q\right)\leq z_{i}^{+}+35,\\ 0, & \mathrm{otherwise}, \end{array}\right.$$

where $M=10^{8}$ is a large penalty coefficient for infeasible regions. The branches in Equation (14) are evaluated from top to bottom in priority order. The method first determines whether the sampling point enters the interior of the cylindrical no-fly zone, then determines whether it is close to the upper or lower cylindrical boundary, and finally determines whether it enters the horizontally expanded buffer zone. The no-fly-zone threat cost of the entire path is

(15) $$J_{2}=\sum_{k=0}^{n}\sum_{l=0}^{N_{s}-1}\sum_{i=1}^{N_{T}}\Gamma_{i}\left(q_{k,l}\right),$$

where $N_{T}$ is the number of no-fly zones. If a sampling point falls inside a no-fly zone, a high-intensity infeasible penalty is imposed. If a sampling point enters the expanded buffer zone or approaches the upper or lower cylindrical boundary, a continuous soft penalty is imposed. A schematic of the threat cost is shown in Figure 1. In the figure, the interior of the no-fly zone corresponds to a high-intensity infeasible penalty, whereas the buffer zone corresponds to a continuous soft penalty.

#### 2.3.3. Terrain Collision and Clearance Function

The terrain collision and clearance term $J_{3}$ simultaneously considers four cases: terrain penetration, flight below the minimum safe clearance, flight below the desired terrain clearance, and excessively high flight. Let the terrain clearance of a sampling point q be

(16) $$\delta \left(q\right)=z\left(q\right)-H\left(x(q),y(q)\right),$$

The minimum safe clearance is $\delta_{\min}=30\;m$, and the desired clearance is $\delta_{\mathrm{opt}}=55\;m$. The point-wise clearance penalty is

(17) $$\Pi_{\delta }\left(q\right)=\left\{\begin{array}{cc} M\exp\left(\min(10,-\delta (q)/8)\right), & \delta (q)<0,\\ 400{\left(\delta_{\min}-\delta \left(q\right)\right)}^{2}, & 0\leq \delta \left(q\right)<\delta_{\min},\\ 0.08\left(\delta_{\mathrm{opt}}-\delta \left(q\right)\right), & \delta_{\min}\leq \delta \left(q\right)<\delta_{\mathrm{opt}},\\ 0.025{\left(\delta \left(q\right)-\delta_{\mathrm{opt}}\right)}^{2}, & \delta \left(q\right)\geq \delta_{\mathrm{opt}}. \end{array}\right.$$

Then

(18) $$J_{3}=\sum_{k=0}^{n}\sum_{l=0}^{N_{s}-1}\Pi_{\delta }\left(q_{k,l}\right).$$

#### 2.3.4. Horizontal Turning-Angle Smoothness Function

The horizontal turning-angle smoothness term $J_{4}$ constrains the turning magnitude of the trajectory on the horizontal plane. Let

(19) $$u_{k}={\left[x_{k+1}-x_{k},\;y_{k+1}-y_{k},\;0\right]}^{T},$$

The horizontal turning angle between two adjacent trajectory segments is

(20) $$\alpha_{k}=\frac{180}{\pi }\arccos\left(\frac{u_{k-1}^{T}u_{k}}{∥u_{k-1}{∥}_{2}{∥u_{k}∥}_{2}}\right),\;k=1,2,\ldots ,n,$$

where $\alpha_{k}$ is measured in degrees. In practical computation, if the length of a horizontal segment is close to 0, the corresponding turning-angle term is skipped. Meanwhile, the ratio inside the arccosine function is clipped to [−1,1] to avoid numerical round-off errors. An additional penalty is imposed for sharp turns exceeding $\alpha_{\max}=45^{\circ }$:

(21) $$J_{4}=\sum_{k=1}^{n}\left[0.15\alpha_{k}^{2}+3\max{\left(0,\alpha_{k}-\alpha_{\max}\right)}^{2}\right].$$

#### 2.3.5. Low-Terrain Guidance Function

To reduce flight risk in complex mountainous environments, this study introduces a low-terrain guidance function to guide trajectories preferentially through relatively low-lying and smooth regions. Let the *k*-th path segment be discretized into $N_{s}$ sampling points with coordinates $q_{k,l}$, and let H(x,y) be the terrain-elevation function. The low-terrain guidance term is defined as

(22) $$J_{5}=\frac{1}{\left(n+1\right)N_{s}}\sum_{k=0}^{n}\sum_{l=0}^{N_{s}-1}H\left(x\left(q_{k,l}\right),y\left(q_{k,l}\right)\right).$$

Because the comprehensive cost function is formulated as a minimization problem, a smaller $J_{5}$ encourages the algorithm to select lower-terrain corridors, thereby reducing the probability that the path crosses high ridges and strongly undulating regions.

#### 2.3.6. Vertical Smoothness Function

To avoid frequent abrupt climbs or descents in the UAV trajectory, this study uses the second-order difference of the altitude sequence to describe vertical smoothness. Let $z_{k}$ denote the altitude of the *k*-th trajectory point. The vertical smoothness cost is defined as

(23) $$J_{6}=\frac{1}{n}\sum_{k=1}^{n}{\left[\left(z_{k+1}-z_{k}\right)-\left(z_{k}-z_{k-1}\right)\right]}^{2}.$$

This term constrains the continuity of altitude variation between adjacent path segments. A smaller $J_{6}$ indicates that the trajectory is smoother in the vertical direction, which helps reduce flight-control difficulty and improve path executability.

#### 2.3.7. Maximum Altitude Constraint Function

Considering that UAV flight missions are typically constrained by airspace altitude and platform performance, this study defines a maximum altitude constraint function. Let the maximum altitude of the flight region be $z_{\max}$, and reserve a safety margin. The altitude upper bound is defined as

(24) $$z_{\mathrm{safe}}=z_{\max}-60,$$

The maximum altitude constraint term is then defined as

(25) $$J_{7}=\sum_{k=0}^{n}\sum_{l=0}^{N_{s}-1}\max{\left(0,z\left(q_{k,l}\right)-z_{\mathrm{safe}}\right)}^{2}.$$

When the altitude of a path sampling point exceeds $z_{\mathrm{safe}}$, this term generates a squared penalty, thereby suppressing excessive climbing and ensuring that the planned path satisfies the maximum flight-altitude constraint.

## 3. TLQ-SGWO Algorithm

### 3.1. Grey Wolf Optimizer

**Figure 2:**
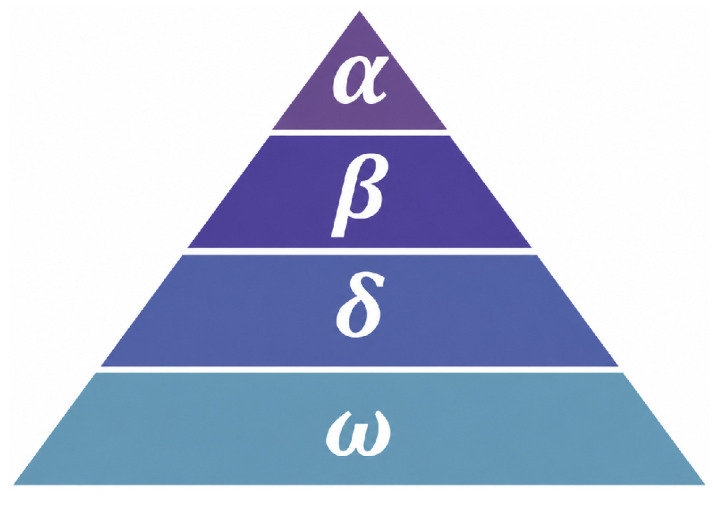
Basic schematic of the grey wolf optimizer.

GWO divides the wolf population into four hierarchical levels, as illustrated in Figure 2. The α wolf acts as the population decision maker, the β wolf assists the α wolf and serves as its candidate, the δ wolf follows the α and β wolves while dominating the remaining individuals, and the ω wolves follow all superior wolves. In the algorithm, these wolves correspond to candidate solutions, and their positions are updated around the α, β, and δ wolves during iteration.

For any variable vector *X*, the standard GWO update is given by

(26) $$\begin{array}{cc} X_{1} & =X_{\alpha }-A_{1}\left|C_{1}X_{\alpha }-X\right|,\\ X_{2} & =X_{\beta }-A_{2}\left|C_{2}X_{\beta }-X\right|,\\ X_{3} & =X_{\delta }-A_{3}\left|C_{3}X_{\delta }-X\right|,\\ X_{new} & =\frac{X_{1}+X_{2}+X_{3}}{3}, \end{array}$$

where

(27) $$A_{i}=2ar_{i}-a,\;C_{i}=2r_{i},\;i=1,2,3,$$

$r_{i}$ is a random vector in [0,1], and *a* is the convergence factor, which decreases linearly from 2 to 0 as the number of iterations increases. $X_{\alpha }$ denotes the position of the population-best solution, namely the α wolf, whereas $X_{\beta }$ and $X_{\delta }$ denote the positions of the β and δ wolves, respectively. In spherical-vector SGWO, the search variables of GWO are defined as three groups of vectors, {ρ,φ,θ}, which are converted into a 3D trajectory by Equation (7) before cost evaluation.

### 3.2. Improved Grey Wolf Optimizer

To address the sensitivity of basic GWO to initial population quality, fixed search-stage transitions, and local stagnation in complex 3D terrain, this study develops TLQ-SGWO within the spherical-vector encoding framework. The main improvements include hybrid initialization and heuristic seed paths, Q-learning-based search-strategy scheduling, Lévy stagnation escape, Alpha local refinement, and a safety-oriented elite branch. These mechanisms retain the leader-wolf update structure of GWO while improving the quality of the initial distribution, adaptive action selection, and controlled handling of stagnation in the cooperative population search process. This design is consistent with the idea of preserving the main optimizer framework while improving strategy adaptivity in reinforcement-learning-assisted and improved GWO studies [18,22].

#### 3.2.1. Hybrid Initialization and Seed Paths

In basic GWO, the population is usually initialized by uniform random sampling. In complex terrain, such initialization may place many individuals in high-penalty terrain or threat regions, weakening early-stage search quality. To reduce this risk, TLQ-SGWO generates 55% of the individuals using a Tent–Logistic hybrid chaotic sequence, while the remaining 45% are generated by uniform random sampling.

The Logistic map is defined as

(28) $$z_{t+1}^{L}=\mu z_{t}^{L}\left(1-z_{t}^{L}\right),\;\mu =4,\;z_{t}^{L}\in \left(0,1\right),$$

The Tent map is defined as

(29) $$z_{t+1}^{T}=\left\{\begin{array}{cc} 2z_{t}^{T}, & 0<z_{t}^{T}<0.5,\\ 2\left(1-z_{t}^{T}\right), & 0.5\leq z_{t}^{T}<1, \end{array}\right.$$

where $z_{0}^{L}$ and $z_{0}^{T}$ are both randomly generated in (0.1,0.9). For the *i*-th chaotic individual and the *j*-th decision dimension, normalized chaotic numbers are generated by alternately using the Logistic map in odd dimensions and the Tent map in even dimensions:

(30) $$\xi_{i,j}=\left\{\begin{array}{cc} z_{i,j}^{L}, & j\;\mathrm{is}\;\mathrm{odd},\\ z_{i,j}^{T}, & j\;\mathrm{is}\;\mathrm{even}, \end{array}\right.$$

Then, $\xi_{i,j}$ is linearly mapped to the spherical-vector variable bounds:

(31) $$\omega_{i,j}=\omega_{j}^{\min}+\xi_{i,j}\left(\omega_{j}^{\max}-\omega_{j}^{\min}\right),$$

where $\omega_{i,j}$ denotes the initial value of the corresponding dimension in ρ, φ, or θ. Alternating the two chaotic maps reduces the distribution bias caused by using a single chaotic map in some dimensions.

In addition to chaotic and random individuals, the algorithm injects three heuristic seed paths. The first seed path follows the straight-line trend from the start point to the goal point. The second slightly increases the pitch angle based on the straight-line trend to provide an initial candidate for crossing terrain undulations. The third introduces a small linear deflection in the heading angle to provide an initial search direction for bypassing threat regions. This design enables the initial population to include both globally dispersed individuals and directionally guided individuals with engineering feasibility, thereby reducing the probability that all candidates fall into high-penalty regions at the initial stage.

#### 3.2.2. Q-Learning Search-Strategy Scheduling

To improve SGWO’s adaptive search capability in complex 3D path planning, this study introduces a Q-learning mechanism for search-strategy scheduling. Q-learning is a model-free reinforcement learning method based on the action-value function [23]. In path planning and metaheuristic optimization, Q-learning and related reinforcement learning mechanisms have been used for hybrid search, strategy selection, and experience learning in swarm intelligence algorithms [22,24,25,26]. In the proposed TLQ-SGWO, Q-learning does not directly generate UAV path-control variables. Instead, it selects different optimizer-level search actions according to the current evolutionary state. The search state consists of the improvement level of the best solution, the population diversity level, and the iteration stage, yielding 3×3×3=27 discrete states.

The action space consists of five search actions, as shown in Table 2. Different actions correspond to local exploitation, standard SGWO update, enhanced exploration, mild Lévy perturbation, and strong Lévy perturbation. With this action design, the algorithm can dynamically adjust its search behavior based on the search state, rather than relying solely on a fixed convergence factor to linearly transition from exploration to exploitation.

The Q-table is updated in Bellman form:

(32) $$Q\left(s_{t},a_{t}\right)\leftarrow Q\left(s_{t},a_{t}\right)+\alpha_{RL}\left[R_{t}+\gamma \max_{a^{\prime }}Q\left(s_{t+1},a^{\prime }\right)-Q\left(s_{t},a_{t}\right)\right],$$

where $\alpha_{RL}$ is the learning rate, γ is the discount factor, and $R_{t}$ is the immediate reward after executing the action. $\alpha_{RL}=0.10$ and γ=0.90. In this study, the reward is primarily determined by the improvement in the best fitness in the current iteration and is further adjusted based on changes in population diversity and the success of Lévy perturbation. When an action effectively reduces the comprehensive cost or improves the search state, a positive reward is assigned. When an action causes stagnation or produces no effective improvement, a smaller reward or penalty is assigned.

Action selection adopts a Boltzmann softmax strategy with temperature decay, where the temperature parameter is denoted as T

(33) $$P\left(a|s\right)=\frac{\exp(q_{a}/T)}{\sum_{j=1}^{5}\exp\left(q_{j}/T\right)}.$$

A higher temperature maintains action exploration in the early stage. As the iterations proceed, the temperature gradually decreases, and the algorithm increasingly favors actions with higher current Q values, thereby improving late-stage search stability.Through this mechanism, Q-learning adaptively schedules the TLQ-SGWO search process based on evolutionary feedback, rather than merely tuning a convergence parameter, thereby improving robustness under complex terrain and no-fly zone constraints.

#### 3.2.3. Lévy Stagnation Escape and α Local Refinement

As the iteration process proceeds, the grey wolf population gradually contracts toward the dominant regions determined by the α, β, and δ wolves. This mechanism improves convergence efficiency. However, in complex mountainous terrain and under multiple no-fly-zone constraints, the search space often exhibits pronounced non-convexity and multimodality, and the population can prematurely aggregate near local feasible corridors or obstacle boundaries, leading to search stagnation. Lévy flights have a long-tailed step-length distribution and are often used for global jumps and local-optimum escape in nature-inspired optimization [27]. Recent optimization studies combining chaotic and Lévy mechanisms also indicate that such perturbations help enhance search diversity and the ability to escape local regions [28]. To alleviate these issues, this study introduces a stagnation-escape mechanism based on Lévy flights and combines it with an α-wolf local- refinement strategy to enhance global escape and late-stage fine search.

Lévy flight is a random search mechanism with a heavy-tailed distribution. Its search process is dominated by small-scale local perturbations while occasionally producing a small number of large-scale jumps. Therefore, this mechanism can maintain local exploitation around the current dominant region while increasing the probability of escaping local optima when stagnation occurs. In this study, the Mantegna algorithm is used to generate Lévy step lengths:

(34) $$L=\frac{u}{{\left|v\right|}^{1/\beta }},\;u∼N\left(0,\sigma_{u}^{2}\right),\;v∼N\left(0,1\right),\;\beta =1.5,$$

where

(35) $$\sigma_{u}={\left[\frac{\Gamma (1+\beta )\sin(\pi \beta /2)}{\Gamma \left(\left(1+\beta \right)/2\right)\beta 2^{(\beta -1)/2}}\right]}^{1/\beta }.$$

Considering that the Lévy distribution may generate excessively large jump lengths, directly applying it to path-encoding variables may disrupt the feasible path structure already obtained. To improve the stability of the perturbation process, this study truncates the Lévy step length as

(36) $$\hat{L}=\mathrm{sign}\left(L\right)\min\left(|L|,3\right).$$

Let the current iteration progress be τ=t/T. TLQ-SGWO does not activate Lévy perturbation when τ<0.45, so as to preserve the natural diffusion of early-stage global search. When τ≥0.45 and the number of consecutive stagnant generations reaches the threshold, Q-learning is allowed to select Lévy actions. Mild Lévy is used for moderate mid-stage stagnation and perturbs approximately 6% of poorer individuals. Strong Lévy is used for more severe late-stage stagnation, perturbs approximately 12% of poorer individuals, and randomly restarts candidate solutions with a small probability. For the selected poorer individuals, the algorithm generates candidates using α as the reference:

(37) $$\begin{array}{cc} \rho^{\prime } & =\rho_{\alpha }+{\hat{L}}_{\rho }\left(\rho^{\max}-\rho^{\min}\right)s_{\rho },\\ \varphi^{\prime } & =\varphi_{\alpha }+{\hat{L}}_{\varphi }s_{\varphi },\\ \theta^{\prime } & =\theta_{\alpha }+{\hat{L}}_{\theta }s_{\theta }, \end{array}$$

where $s_{\rho }$, $s_{\varphi }$, and $s_{\theta }$ are perturbation scales set according to the iteration stage. After boundary repair and Cartesian path conversion, the candidate solution is re-evaluated, and it is retained only when it is better than the original poorer individual. Thus, the Lévy module performs directed escape after stagnation rather than unconditionally increasing randomness.

α local refinement is used to compensate for the insufficient fine exploitation of Lévy long jumps and GWO population updates in the late stage. When Q-learning selects the exploit action, or when the iteration progress exceeds 0.82, the algorithm generates several Gaussian-neighborhood candidates around the current α:

(38) $$\begin{array}{cc} \rho^{\prime } & =\rho_{\alpha }+N\left(0,\sigma_{\rho }^{2}\right),\\ \varphi^{\prime } & =\varphi_{\alpha }+N\left(0,\sigma_{a}^{2}\right),\\ \theta^{\prime } & =\theta_{\alpha }+N\left(0,\sigma_{a}^{2}\right), \end{array}\;$$

where

(39) $$\sigma_{\rho }=\left(\rho^{\max}-\rho^{\min}\right)\left[0.050(1-\tau )+0.004\right],\;\sigma_{a}=0.070\left(1-\tau \right)+0.004.$$

As τ increases, the perturbation scale gradually decreases, allowing the search to transition from mid-stage neighborhood probing to late-stage small-range refinement. If a local candidate path obtains a lower cost, α, β, and δ are updated immediately. In coordination with the exploit action of Q-learning, this mechanism enables the algorithm to continue reducing path cost, improving smoothness, and decreasing altitude redundancy after a feasible corridor is found, thereby improving the quality of the final path solution.

Therefore, Lévy stagnation escape mainly enhances the algorithm’s ability to escape local feasible corridors under complex constraints, whereas local refinement mainly improves late-stage search accuracy around the current best solution. Their complementary roles allow TLQ-SGWO to combine controlled escape with fine trajectory refinement, thereby improving search stability and path quality in complex 3D path-planning problems.

### 3.3. Complexity Analysis

Let the population size be $N_{p}$, the number of trajectory segments be *n*, the number of sampling points per segment be $N_{s}$, and the maximum number of iterations be *T*. The main cost of one path evaluation comes from sampling each path segment and calculating the threat and terrain costs, with a complexity of approximately $O(nN_{s})$. Therefore, the time complexity of the main loop is

(40) $$O(TN_{p}nN_{s}).$$

The Q-learning module only maintains a 27×5 Q-table, and its computational overhead is negligible relative to path evaluation. If $R_{s}$ safety branches are enabled, the additional complexity is approximately $O(R_{s}TN_{p}^{\prime }nN_{s})$, where $N_{p}^{\prime }\approx 0.6N_{p}$. The space complexity is mainly determined by the population and Q-table, namely $O(N_{p}n+27\times 5)$.

### 3.4. TLQ-SGWO Algorithm Procedure

The novelty of TLQ-SGWO does not lie in the spherical-vector representation itself or in the conventional leader-based SGWO update mechanism. Instead, it lies in embedding a state-dependent strategy scheduler into the spherical-vector SGWO framework. In the proposed method, the search state is characterized by the current improvement level, population diversity, and evolutionary phase. Based on this state information, Q-learning is used to adaptively select among exploitation, standard search, enhanced exploration, and mild or strong stagnation-escape actions. The Tent–Logistic hybrid initialization improves the diversity and ergodicity of the initial population, whereas Lévy perturbation and Alpha refinement are conditionally activated according to the selected search action and stagnation condition. Therefore, the proposed contribution is a coordinated adaptive search framework for constrained 3D trajectory optimization, rather than a new trajectory-encoding scheme alone.

Algorithm 1 summarizes the complete optimization procedure of TLQ-SGWO. The inputs include the terrain model M, the comprehensive cost function *J*, the population size $N_{p}$, and the maximum number of iterations *T*. The outputs are the best Cartesian path and the corresponding best-cost curve. The procedure integrates spherical-vector population initialization, state-dependent action selection, leader-based population updating, conditional stagnation escape, and local refinement within a unified SGWO framework.

Algorithm 1 begins by generating a mixed population in the spherical-vector decision space and replacing three individuals with heuristic seed paths. After all candidates are decoded into Cartesian trajectories and evaluated by the seven-term cost function, the three leader wolves are identified and the 27×5 Q-table is initialized. At iteration *t*, the relative improvement of the best cost, the population diversity relative to its moving baseline, and the normalized iteration phase are each quantized into three levels, producing one of 27 discrete search states. Specifically, the improvement thresholds are $10^{-5}$ and $10^{-2}$, the diversity-ratio thresholds are 0.5 and 1.2, and the iteration phases are divided at t/T=0.33 and t/T=0.66.

For the current state, an action is sampled using the Boltzmann softmax policy. The temperature decays from $T_{\mathrm{init}}=1.30$ to $T_{\mathrm{final}}=0.25$ according to

(41) $$T_{t}=T_{\mathrm{init}}{\left(\frac{T_{\mathrm{final}}}{T_{\mathrm{init}}}\right)}^{t/T}.$$

The selected action adjusts the SGWO control parameter and determines whether standard updating, enhanced exploration, Lévy-based escape, or local exploitation is emphasized. The standard SGWO update is then applied to (ρ,φ,θ) for every wolf. Each updated solution is repaired to satisfy the variable bounds, decoded into a Cartesian trajectory, evaluated by *J*, and used to update the three leader wolves.
**Algorithm 1** TLQ-SGWO for UAV 3D Path Planning.  1:**Input:** terrain model M, cost function *J*, population size $N_{p}$, max iteration *T*  2:Generate 55% chaos-based wolves and 45% random wolves  3:Inject three heuristic seeded paths  4:Evaluate all wolves and identify α, β, and δ  5:Initialize $Q\in R^{27\times 5}$  6:**for** t=1 to *T* **do**  7:      Encode state $s_{t}$ using improvement, diversity and phase  8:      Select action $a_{t}$ by Boltzmann softmax on $Q(s_{t},\cdot )$  9:      Set GWO control parameter according to the selected action10:      **for** each wolf **do**11:           Update (ρ,φ,θ) by SGWO rule12:           Repair bounds, convert to Cartesian path and evaluate *J*13:           Update α, β, and δ14:      **end for**15:      **if** $a_{t}$ is Lévy_mild or Lévy_strong **then**16:           Apply controlled Lévy perturbation to poor wolves17:      **end if**18:      **if** $a_{t}$ is exploit or t/T>0.82 **then**19:           Refine α by local Gaussian perturbation20:      **end if**21:      Compute reward and update Q-table22:      Record best cost23:**end for**24:**if** safety branch is enabled **then**25:      Run independent safety SGWO restarts and keep the better result26:**end if**27:**Output:** best Cartesian path and best-cost curve

The two Lévy actions are disabled before 45% of the iterations and are activated only after the corresponding stagnation threshold is reached. Mild and strong Lévy perturbations are applied to approximately 6% and 12% of the poorer wolves, respectively. When the exploitation action is selected, or when t/T>0.82, Gaussian-neighborhood candidates are generated around the current Alpha wolf for local refinement. The immediate reward is calculated from the relative best-cost improvement and is adjusted according to the diversity change and the success of stagnation escape. The Q-table is subsequently updated with learning rate $\alpha_{RL}=0.10$ and discount factor γ=0.90. Table 3 summarizes the principal thresholds used in the procedure.

After the main loop terminates, the best spherical-vector solution is converted into the final Cartesian path. Therefore, TLQ-SGWO forms a closed-loop adaptive optimization procedure in which the current search state guides the selection of search actions, and the resulting search performance further updates the Q-table for subsequent iterations.

## 4. Simulation Experiments and Result Analysis

### 4.1. Experimental Settings

To evaluate TLQ-SGWO from both general optimization and task-specific path-planning perspectives, the experiments are organized into two levels. The first level uses CEC2017 benchmark-function testing to examine general continuous optimization capability. The second level uses artificial complex mountainous path-planning experiments to evaluate constrained UAV 3D path-planning performance. The seven evaluated algorithms are GWO, SGWO, TLQ-SGWO, WOA, GA, PSO, and RCGA-PSO. GWO provides the basic optimizer baseline, whereas SGWO is the closest spherical-vector predecessor of the proposed method. WOA and PSO represent widely used swarm-intelligence optimizers, and GA represents a classical evolutionary-search paradigm. RCGA-PSO is implemented following the established GA–PSO hybridization principle, which combines PSO-based individual and social learning with real-coded crossover and mutation operations [29]; it is selected to examine whether the proposed method remains competitive against a stronger hybrid evolutionary-search mechanism. Together, these methods enable comparisons with baseline, closely related, mainstream, evolutionary, and hybrid approaches. MATLAB R2024b (MathWorks, Natick, MA, USA) is used as the simulation tool. The experimental environment is a computer equipped with an Intel(R) Core(TM) i7-11600H processor and 16 GB RAM, running the Windows 11 operating system (64-bit).

The CEC2017 test uses the official evaluator and its shift, rotation, and permutation data. The test functions are F1–F30, and the problem dimension is set to D=30. To ensure fair comparison, all algorithms use the same function-evaluation budget for each function and each independent run, namely MaxFEs=10,000D=300,000. The population size is $N_{p}=30$, and each algorithm is independently run 30 times. The CEC2017 test examines the general continuous optimization capability of the algorithms and does not include UAV terrain, no-fly-zone, or trajectory-smoothness constraints.

The artificial terrain experiments use artificial complex mountainous terrain. The map size is 1000m×1000m, and the maximum altitude is 500m. These experiments are designed to systematically compare path-planning performance under controllable numbers of no-fly zones. The number of path nodes is set to n=12. All algorithms use the same cost-function weights and the same start and goal points. The parameters for the artificial terrain comparison experiments are T=300, $N_{p}=300$, and 15 independent runs. The complete TLQ-SGWO configuration incorporates the main optimization procedure together with additional safety-oriented search mechanisms to further improve path feasibility in complex constrained environments. For computational-cost evaluation, the reported runtime focuses on the main TLQ-SGWO optimization process under the same evaluation setting, including hybrid initialization, Q-learning-based strategy scheduling, L’evy stagnation escape, and Alpha refinement, so that the time comparison reflects the computational burden of the core proposed method.

### 4.2. CEC2017 Benchmark-Function Testing

To examine the general continuous optimization capability of TLQ-SGWO, this study first evaluates the algorithm on the CEC2017 benchmark suite before conducting the artificial terrain path-planning experiments. CEC2017 contains test functions with different landscape characteristics, including unimodal, multimodal, hybrid, and composition functions, as summarized in Table 4. Therefore, it can be used to evaluate global exploration, local exploitation, and convergence stability under different levels of optimization difficulty [30]. This study uses the official CEC2017 evaluator and its shift, rotation, and permutation data. The test functions are F1–F30, the search range is ${\left[-100,100\right]}^{D}$, and the problem dimension is set to D=30. The official bias value is removed from the final objective value, and the error value is used as the evaluation metric:

(42) $${\mathrm{Error}}_{i}=\max\left(0,f_{i}\left(x\right)-f_{i}^{*}\right),\;f_{i}^{*}=100i,$$

where $f_{i}^{*}$ is the official optimal bias value of the *i*-th CEC2017 function.

The compared algorithms include GWO, SGWO, WOA, GA, PSO, RCGA-PSO, and TLQ-SGWO. To ensure a fair comparison, all algorithms use the same function-evaluation budget for each function and each independent run, namely MaxFEs=10,000D=300,000. The population size is set to $N_{p}=30$, and each algorithm is independently run 30 times. Table 5 reports the mean error (Mean) and standard deviation (Std) obtained by each algorithm on each function. Since all values are error values after removing the official bias, smaller values indicate better performance. The best result in each Mean or Std row is highlighted in bold.

Because the numerical scales of different CEC2017 functions vary significantly, the detailed Mean/Std table is further summarized using ranking-based indicators. For each function, algorithms are ranked according to their Mean and Std values, respectively, and a smaller rank indicates better performance. Table 6 reports the average ranking and the number of first-place results over the 30 functions.

As shown in Table 6, TLQ-SGWO obtains the best average rank in terms of both Mean and Std. Although PSO and RCGA-PSO obtain the largest numbers of best Mean values on some individual functions, TLQ-SGWO provides the most balanced overall performance over the entire benchmark suite. In particular, the best Std rank and the largest Best Std count indicate that TLQ-SGWO has stronger stability across independent runs. This result is consistent with the design motivation of the proposed method: Tent–Logistic initialization improves initial population diversity, while Q-learning-based strategy scheduling adaptively balances exploration and exploitation during the search process.

To further evaluate whether the proposed mechanisms effectively improve the original grey-wolf-based framework, Table 7 summarizes the pairwise comparison between TLQ-SGWO and each competing algorithm. A larger count indicates that TLQ-SGWO obtains a lower Mean or Std value on more benchmark functions.

The pairwise comparison shows that TLQ-SGWO achieves lower Mean errors than both GWO and SGWO on all 30 functions. This is an important result because TLQ-SGWO is developed from the grey wolf optimization framework. The consistent improvement over GWO and SGWO indicates that the proposed grey-wolf-based adaptive search framework, Tent–Logistic initialization, and Q-learning-driven strategy scheduling substantially enhance the search capability of the original framework. TLQ-SGWO also outperforms WOA and GA on most functions and remains competitive against PSO and RCGA-PSO. Although TLQ-SGWO is not uniformly dominant on every single function, it shows a more robust and balanced performance distribution.

Table 8 further reports the average Mean-error rank on different types of CEC2017 functions. This grouped comparison helps identify the type of search landscape on which each algorithm performs better.

The grouped ranking results show that TLQ-SGWO has its most evident advantage on the composition functions F21–F30, where it achieves the best average rank of 1.50. Composition functions usually contain more complicated landscape characteristics and stronger interactions among different search components. Therefore, this result suggests that TLQ-SGWO has better adaptability to complex multimodal optimization problems. This property is highly relevant to UAV three-dimensional path planning, because the path-planning problem considered in this study is also nonlinear, constrained, and multimodal. Overall, the CEC2017 results demonstrate that TLQ-SGWO does not merely improve several isolated functions, but provides a consistent improvement over the original GWO and SGWO and achieves strong overall robustness among the compared algorithms.

### 4.3. Artificial Terrain Scenario Settings

The evaluation scenarios consist of four artificial terrain test scenarios with different levels of difficulty. The four test scenarios differ in the number of cylindrical no-fly zones, while the terrain seed, start and goal points, number of path nodes, and cost-function weights remain the same. Specifically, Scenario 1, Scenario 2, Scenario 3, and Scenario 4 contain 2, 3, 4, and 5 cylindrical no-fly zones, corresponding to NF1–NF2, NF1–NF3, NF1–NF4, and NF1–NF5, respectively.

Each no-fly zone is described by the horizontal center coordinates $\left(x_{i},y_{i}\right)$, bottom altitude $z_{i}^{\min}$, radius $r_{i}$, vertical height $h_{i}$, top altitude $z_{i}^{\max}$, and threat intensity $\lambda_{i}$. The specific parameters are listed in Table 9. Here, $z_{i}^{\max}$ does not exceed the maximum flight altitude of 500m.

### 4.4. Algorithm Comparison Experiments

Table 10 summarizes the artificial mountainous path-planning results of the seven algorithms in the four scenarios with 2–5 cylindrical no-fly zones. The terrain seed, start and goal points, number of path nodes, and cost-function weights are fixed, whereas the number of no-fly zones is gradually increased to raise threat density and constraint complexity. Overall, TLQ-SGWO shows the most stable performance trend across the four scenarios. RCGA-PSO is also a strong competitor, especially in the lower-complexity setting, but its overall performance remains weaker than that of TLQ-SGWO as the scenarios become more constrained.

In Scenario 1, the threat density is relatively low, and TLQ-SGWO and RCGA-PSO form the leading group. RCGA-PSO achieves the lowest mean cost of 3306.63, whereas TLQ-SGWO yields a nearly identical mean cost of 3316.58. The difference between the two methods is not statistically significant (p=0.3837). However, TLQ-SGWO attains the smallest standard deviation, 313.22, indicating more stable performance across independent runs. This result suggests that when the feasible corridor is still relatively wide, several strong optimizers can identify comparable low-cost routes, and the advantage of TLQ-SGWO is not yet strongly separated from that of RCGA-PSO.

As the number of cylindrical no-fly zones increases, the advantage of TLQ-SGWO becomes clearer. In Scenario 2, TLQ-SGWO achieves the lowest mean cost of 3728.22, whereas RCGA-PSO increases to 6017.83. In Scenario 3, TLQ-SGWO again yields the lowest mean cost, 5542.61, compared with 6590.84 for RCGA-PSO. In Scenario 4, TLQ-SGWO maintains the lowest mean cost, 5269.77, while RCGA-PSO gives 6004.86. These results indicate that TLQ-SGWO adapts more effectively to the progressive increase in threat density and path-planning complexity. This improvement may be attributed to the combined effect of improved initial population quality, Q-learning-based search-strategy scheduling, and controlled stagnation escape, which help the population avoid high-penalty regions and refine feasible trajectories in the middle and late stages.

The computational time of each algorithm is measured using MATLAB’s tic and toc functions. The timing interval covers only the optimization process and excludes terrain construction, post-optimization path-length statistics, statistical post-processing, figure generation, and file input/output. As reported in Table 11, GA and RCGA-PSO have the lowest overall mean runtimes of 11.730 and 11.893 s, respectively, followed by TLQ-SGWO with an overall mean runtime of 12.973 s. The corresponding overall mean runtimes of GWO, SGWO, WOA, and PSO are 13.164, 14.675, 13.475, and 17.921 s, respectively. Therefore, TLQ-SGWO is not the fastest algorithm in terms of pure runtime, but its computational cost remains competitive among the compared methods. Its Q-table update, strategy-selection operation, chaotic initialization, and controlled stagnation escape introduce only modest overhead relative to repeated path-cost evaluations.

More importantly, this slight increase in runtime is accompanied by a clear improvement in path-planning performance. Compared with RCGA-PSO, TLQ-SGWO increases the overall mean runtime by only about 1.08 s, corresponding to approximately 9.1%. Compared with GA, the increase is about 1.24 s, corresponding to approximately 10.6%. However, in the more complex scenarios, TLQ-SGWO achieves substantially lower path costs. Taking RCGA-PSO as a representative competitive hybrid algorithm, TLQ-SGWO reduces the mean path cost by approximately 38.0%, 15.9%, and 12.2% in Scenarios 2, 3, and 4, respectively. These results indicate that the proposed improvement mechanisms do not simply increase computational complexity, but effectively convert a small amount of additional computation into better feasible-trajectory refinement and stronger avoidance of high-penalty regions.

In addition, TLQ-SGWO exhibits the smallest overall runtime standard deviation, 0.407 s, indicating more stable computational behavior across different scenarios. Its mean runtimes in Scenarios 1–4 are 13.020, 12.682, 13.062, and 13.130 s, respectively, showing that the runtime does not increase significantly as the number of cylindrical no-fly zones increases. Therefore, TLQ-SGWO achieves a favorable balance between solution quality, path feasibility, and computational efficiency. However, the average running time of approximately 13 seconds also indicates that the current MATLAB implementation is more suitable for offline or pre-task drone path planning.

**Figure 3:**
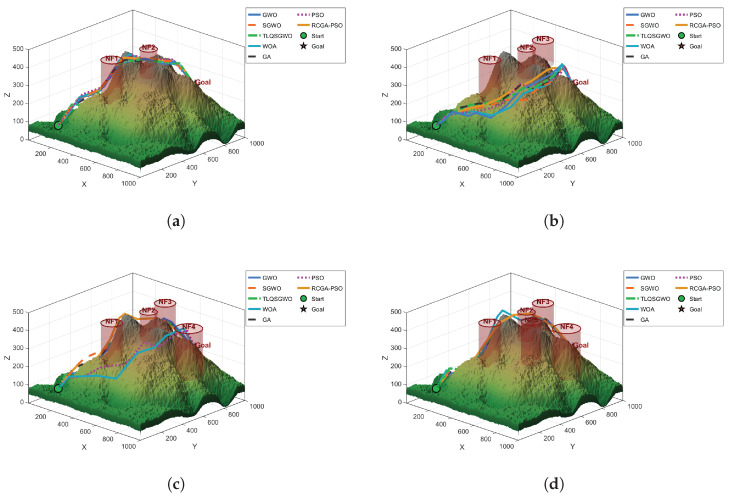
3D comparison of the best paths obtained by the seven algorithms in four scenarios. Path colors identify the algorithms as shown in each legend; the green circle and red star denote the start and goal, respectively, and the translucent red cylinders denote no-fly zones. (**a**) Scenario 1: 2 cylindrical no-fly zones; (**b**) Scenario 2: 3 cylindrical no-fly zones; (**c**) Scenario 3: 4 cylindrical no-fly zones; (**d**) Scenario 4: 5 cylindrical no-fly zones.

Figure 3 shows the best 3D paths generated by the seven algorithms in the four scenarios. All algorithms can produce feasible trajectories, but their path quality becomes increasingly differentiated as the no-fly-zone configuration becomes denser. In Scenario 1, TLQ-SGWO and RCGA-PSO both bypass the threat regions effectively and maintain relatively smooth routes, which agrees with their close mean-cost results. From Scenario 2 onward, however, TLQ-SGWO more consistently preserves smoother and lower-cost trajectories in the presence of narrower feasible corridors and stronger terrain–threat coupling. This behavior suggests that the proposed method maintains more effective search quality when the path-planning environment becomes more constrained.

**Figure 4:**
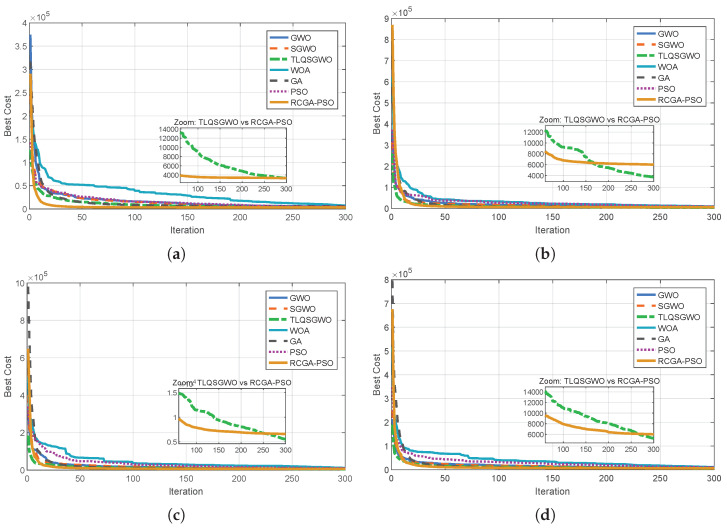
Comparison of average fitness convergence curves of different algorithms under scenarios with different complexity levels. (**a**) Scenario 1: 2 cylindrical no-fly zones; (**b**) Scenario 2: 3 cylindrical no-fly zones; (**c**) Scenario 3: 4 cylindrical no-fly zones; (**d**) Scenario 4: 5 cylindrical no-fly zones.

Figure 4 shows the average convergence curves of the seven algorithms in the four scenarios. In Scenario 1, the curves of TLQ-SGWO and RCGA-PSO remain close in the middle and late stages, which is consistent with the near tie in their final mean costs. As the number of cylindrical no-fly zones increases from Scenario 2 to Scenario 4, TLQ-SGWO maintains a lower middle-to-late-stage fitness level than the comparison algorithms, including RCGA-PSO, and its advantage becomes visually clearer in the enlarged convergence view. This result suggests that the proposed method provides stronger robustness under increasing constraint complexity. The continued late-stage decrease also indicates that Q-learning-based strategy scheduling, Lévy stagnation escape, and α local refinement jointly contribute to local-region escape and late-stage trajectory refinement.

To assess whether the observed final-cost differences are statistically reliable, nonparametric statistical tests are conducted based on 15 independent runs. The Friedman test is first used to examine the overall performance differences among the seven algorithms in each scenario. Then, with TLQ-SGWO as the reference, the Wilcoxon rank-sum test is used for pairwise comparisons between TLQ-SGWO and each comparison algorithm. The significance level is set to α=0.05.

The Friedman test yields $p=3.014\times 10^{-11}$, $3.132\times 10^{-6}$, $2.263\times 10^{-7}$, and $7.578\times 10^{-11}$ for Scenario 1 to Scenario 4, respectively, indicating significant overall differences in final cost in all four scenarios. Pairwise Wilcoxon rank-sum tests show that TLQ-SGWO is significantly better than GWO, SGWO, WOA, GA, and PSO in every scenario. With respect to RCGA-PSO, the difference is not significant in Scenario 1 (p=0.3837), but becomes significant in Scenario 2 (p=0.004795), Scenario 3 (p=0.01440), and Scenario 4 (p=0.01140). These results support the interpretation that RCGA-PSO is a strong comparison method under relatively mild constraints, whereas TLQ-SGWO shows clearer advantages when threat density and constraint complexity increase.

### 4.5. Ablation Study

To analyze the contribution of each module, this study adopts a two-level ablation design. The ablation study is conducted on Scenario 3 with four cylindrical no-fly zones, which represents a medium-to-high constraint-density environment. First, five equal-budget core ablation schemes, A1–A5, are configured, all with T=300, $N_{p}=400$, and 20 independent runs. A1 is the basic spherical-vector grey wolf optimizer and retains only the main SGWO search. A2 adds Tent–Logistic hybrid initialization and heuristic seed paths to A1. A3 further introduces Q-learning search-strategy scheduling but does not enable Lévy perturbation, Alpha refinement, or the safety-oriented elite branch. A4 adds stagnation-triggered Lévy perturbation to A3. A5 adds conservative terminal Alpha local refinement to A4. Second, to evaluate the robust output capability of the complete TLQ-SGWO in offline safety planning, A6 further enables the safety-oriented elite branch on the basis of A5. Because A6 includes additional safety-redundancy search, its computational budget is higher than those of A1–A5. Therefore, A6 is not used for strict equal-budget module attribution, but is interpreted as supplementary robustness validation of the complete algorithm. The results are shown in Table 12.

**Figure 5:**
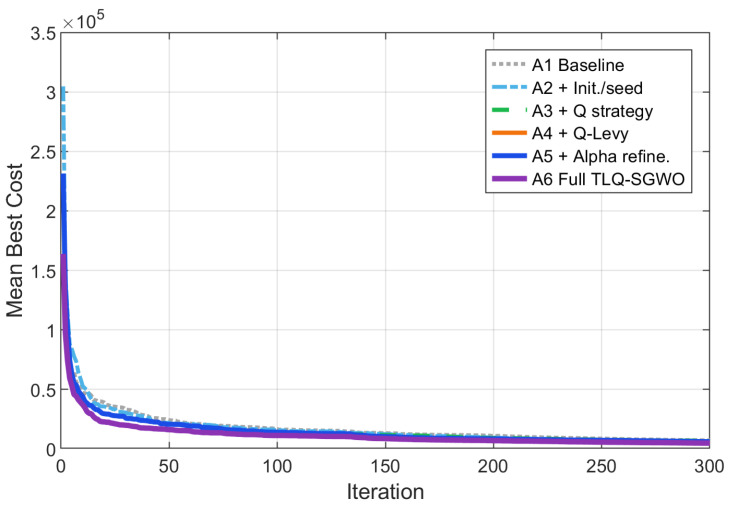
Average convergence curves in the ablation study.

**Figure 6:**
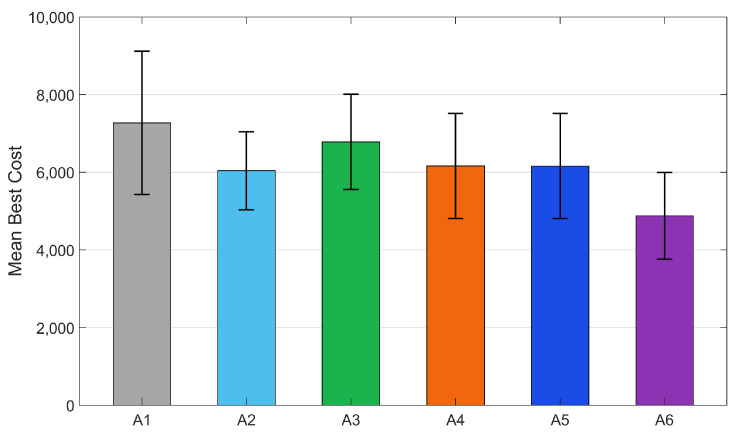
Cost analysis of the ablation study.

Figure 5 and Figure 6 respectively present the convergence trends and mean-cost comparison of the ablation schemes.

Figure 5 shows the average convergence trends of the ablation schemes over 20 independent runs. A1–A5 are used to examine the stepwise effects of the equal-budget core modules. Compared with A1, the mean cost of A2 decreases from 7272.32 to 6040.75, corresponding to a reduction of 1231.56 and 16.9%, indicating that hybrid initialization and heuristic seed paths provide the most evident average-cost reduction in this scenario. After Q-learning strategy scheduling is added alone in A3, the mean cost increases to 6784.04, suggesting that adaptive strategy switching may introduce additional search fluctuations when it is not coordinated with stagnation-escape operations. After Lévy perturbation is added to A3, the mean cost of A4 decreases to 6159.45 and the best cost decreases to 3885.69, indicating that stagnation-triggered Lévy perturbation can partially compensate for the search fluctuation introduced by strategy scheduling and improve the ability to discover lower-cost solutions. However, A4 and A5 still do not outperform A2 in terms of mean cost under the equal-budget setting, so these results should not be interpreted as monotonic improvement from every added module. The terminal Alpha refinement in A5 produces only a 0.54 reduction in mean cost, suggesting that it mainly acts as a conservative fine-tuning mechanism with limited independent contribution to average-cost reduction in the current experiment. After the additional safety-oriented elite branch is enabled in A6, the mean cost decreases to 4877.74, showing that the complete TLQ-SGWO further improves robust output quality under an offline planning setting that allows an additional safety-redundancy search budget. Because the benefit of A6 includes both the safety-branch mechanism and the additional search budget, A6 is not used for strict equal-budget attribution, but is treated as robustness validation of the complete algorithm.

Stepwise Wilcoxon signed-rank paired tests show that the improvement from A1 to A2 is statistically significant (p=0.0132), and the improvement from A3 to A4 is also statistically significant (p=0.0072). The transitions from A2 to A3 and from A4 to A5 do not reach statistical significance in the current batch. A Friedman overall test is conducted on the equal-budget core ablation schemes A1–A5, yielding $\chi^{2}=8.5445$ and p=0.07355. Because this result does not reach the 0.05 significance level, the equal-budget ablation provides only partial statistical support for the differences among the core-module combinations. The difference between A6 and A5 is not interpreted as an equal-budget ablation, but is used to show that the complete algorithm has stronger robustness under an additional safety-redundancy budget. Overall, the ablation results partially support the effectiveness of the proposed modules: hybrid initialization and seed paths make the clearest contribution to average-cost reduction, Q-learning appears more useful when coupled with Lévy stagnation escape than when used alone, Alpha terminal refinement mainly plays a conservative fine-tuning role, and the safety-oriented elite branch improves robust output quality under an additional search budget.

## 5. Conclusions

To address UAV path planning in complex 3D terrain with terrain, no-fly-zone, clearance, smoothness, and altitude constraints, this study proposes TLQ-SGWO, a Tent–Logistic- and Q-learning-based multi-strategy spherical-vector grey wolf optimizer. The proposed method encodes trajectory segments using spherical vector increments and formulates 3D path planning as a multi-constraint cost-minimization problem via a seven-term comprehensive cost function.

The algorithm improves the initial distribution through Tent–Logistic hybrid initialization and heuristic seed paths, uses Q-learning to schedule local exploitation, standard search, enhanced exploration, and Lévy escape actions, and combines Alpha local refinement with a safety-oriented elite branch to improve feasible-solution quality in the later search stage.

The CEC2017 benchmark results show that TLQ-SGWO achieves the best overall average rankings in both mean error and standard deviation among the seven compared algorithms. Although PSO and RCGA-PSO obtain the best mean values on more individual functions, TLQ-SGWO provides a more balanced performance distribution across the whole benchmark suite and consistently outperforms the original GWO and SGWO on all 30 functions. These results indicate that the proposed adaptive search framework effectively improves the original grey-wolf-based optimizer while maintaining strong robustness across different function landscapes. In the artificial mountainous scenarios with 2–5 cylindrical no-fly zones, TLQ-SGWO achieves the lowest mean cost in Scenario 2 to Scenario 4 and remains statistically comparable to RCGA-PSO in Scenario 1 while yielding the smallest standard deviation. Statistical tests support the reliability of the observed final-cost differences. The ablation study indicates that Q-learning-based strategy scheduling is most effective when coupled with stagnation-escape operations, suggesting that adaptive strategy selection should be coordinated with diversity-restoration mechanisms. Hybrid initialization and seed paths provide the most evident average-cost reduction, while Alpha terminal refinement mainly serves as a conservative fine-tuning mechanism. Under an additional safety-redundancy budget, the complete TLQ-SGWO reduces the average cost by 32.9% compared with the baseline SGWO. With the optional safety-redundancy branch disabled, its overall mean runtime in the artificial-terrain experiments is 12.973 s, compared with 14.675 s for SGWO. This result indicates that the core adaptive search mechanisms do not impose excessive computational overhead, although the current implementation remains primarily intended for offline or pre-mission planning.

The current experiments are mainly based on artificial terrain, fixed start and goal points, and static cylindrical no-fly zones, and they do not yet systematically cover dynamic obstacles, realistic flight dynamics, or real-flight validation. Dynamic urban environments usually require both offline global search and online replanning [31]. The weights of the cost function are also manually specified; future work may combine multi-objective multi-task optimization with source-task transfer to reduce dependence on manual parameter tuning [32]. If the method is extended to multi-UAV cooperative tasks, event-triggered formation control, communication delays, cooperative risk perception, and complex map structure representation should also be considered [33,34,35]. Future research will extend the method to broader environmental and task scales, investigate adaptive weighting or multi-objective optimization, and verify the executability of trajectories under realistic UAV dynamic constraints. In future work, field experiments will be conducted to further validate the algorithm under real-world terrains and non-cylindrical, irregularly shaped no-fly zones.

## Figures and Tables

**Table 1:** Cost terms and weights.

Cost Term	Physical Meaning	Weight
$J_{1}$	3D path length	$b_{1}=1.00$
$J_{2}$	Cylindrical no-fly-zone threat penalty	$b_{2}=8.00$
$J_{3}$	Terrain collision and clearance penalty	$b_{3}=3.00$
$J_{4}$	Horizontal turning-angle smoothness cost with an enhanced penalty above $45^{\circ }$	$b_{4}=15.00$
$J_{5}$	Low-terrain corridor guidance cost	$b_{5}=5.00$
$J_{6}$	Vertical smoothness cost	$b_{6}=0.05$
$J_{7}$	Maximum safe-altitude constraint	$b_{7}=1.00$

**Table 2:** Q-learning action space.

Action Index	Code Name	Functional Description
1	exploit	Strengthen local exploitation and trigger Alpha local refinement
2	normal	Standard SGWO search
3	explore	Increase exploration intensity and expand the search range
4	Lévy_mild	Apply mild Lévy perturbation to poorer individuals after stagnation
5	Lévy_strong	Apply strong Lévy perturbation or partial restart under severe stagnation

**Table 3:** Principal parameters and thresholds in the TLQ-SGWO procedure.

Component	Setting	Function in the Procedure
State: improvement	$10^{-5}$, $10^{-2}$	Quantizes the relative best-cost improvement into low, medium, and high levels
State: diversity ratio	0.5, 1.2	Quantizes current diversity relative to its moving baseline
State: iteration phase	0.33, 0.66	Divides the search into early, middle, and late stages
Q-learning	$\alpha_{RL}=0.10$, γ=0.90	Controls Q-value learning and future-reward discounting
Softmax temperature	1.30 → 0.25	Gradually shifts action selection from exploration toward learned preferences
Lévy activation	t/T≥0.45	Prevents premature perturbation during early population diffusion
Lévy population ratios	6%, 12%	Perturbs poorer wolves under mild and strong escape actions, respectively
Alpha refinement	exploitation action or t/T>0.82	Activates Gaussian-neighborhood search around the current best solution

**Table 4:** Grouping of CEC2017 test functions.

Function Category	Function Index	Dimension	Search Range
Unimodal functions	F1–F3	30	[−100,100]
Multimodal functions	F4–F10	30	[−100,100]
Hybrid functions	F11–F20	30	[−100,100]
Composition functions	F21–F30	30	[−100,100]

**Table 5:** Experimental results on CEC2017 test functions (D=30; best values in each row are shown in bold).

Function	Metric	GWO	SGWO	TLQ-SGWO	WOA	GA	PSO	RCGA-PSO
F1	Mean	$1.25\times 10^{10}$	$1.27\times 10^{10}$	$1.38\times 10^{6}$	$7.54\times 10^{7}$	$3.28\times 10^{9}$	$9.26\times 10^{8}$	** 5759.64 **
	Std	$1.71\times 10^{9}$	$1.71\times 10^{9}$	$5.79\times 10^{5}$	$1.04\times 10^{8}$	$1.62\times 10^{9}$	$1.22\times 10^{9}$	** 5440.35 **
F2	Mean	$1.28\times 10^{37}$	$6.74\times 10^{36}$	$7.66\times 10^{7}$	$1.25\times 10^{24}$	$2.12\times 10^{35}$	$5.98\times 10^{34}$	** $5.97\times 10^{4}$ **
	Std	$2.81\times 10^{37}$	$2.07\times 10^{37}$	$2.58\times 10^{8}$	$4.76\times 10^{24}$	$9.38\times 10^{35}$	$3.27\times 10^{35}$	** $3.25\times 10^{5}$ **
F3	Mean	$9.07\times 10^{4}$	$8.91\times 10^{4}$	$4.55\times 10^{4}$	$2.90\times 10^{4}$	$8.10\times 10^{4}$	5531.34	** 3738.64 **
	Std	4997.75	6077.75	8062.64	8154.69	$3.26\times 10^{4}$	8799.09	** 4108.59 **
F4	Mean	725.67	721.13	** 72.51 **	129.23	295.26	209.69	86.43
	Std	174.61	251.22	** 1.81 **	58.76	125.29	126.42	29.62
F5	Mean	333.18	320.90	157.23	242.09	134.00	** 81.24 **	126.39
	Std	26.90	23.24	** 19.99 **	72.11	33.26	21.03	41.53
F6	Mean	49.63	52.27	14.48	67.24	5.34	2.62	** 0.01 **
	Std	8.18	7.92	13.59	12.24	2.50	2.08	** $6.42\times 10^{-3}$ **
F7	Mean	402.33	392.06	165.39	362.31	250.57	** 104.69 **	148.42
	Std	41.55	40.27	38.17	70.76	50.02	** 18.85 **	29.35
F8	Mean	238.08	239.28	100.90	199.71	125.34	** 75.56 **	122.24
	Std	22.50	26.19	** 13.12 **	45.05	23.72	18.87	31.14
F9	Mean	5125.80	5624.78	262.70	6959.75	2959.45	** 158.05 **	2122.77
	Std	1739.48	2007.15	781.80	2717.98	1605.38	** 235.20 **	1451.54
F10	Mean	6255.87	6216.61	3824.35	4933.31	3164.65	3057.37	** 2959.64 **
	Std	472.70	519.65	** 435.17 **	775.31	547.05	623.63	540.39
F11	Mean	9508.45	9886.21	** 101.24 **	328.61	7369.48	210.11	166.13
	Std	239.08	1408.05	** 32.07 **	100.85	5506.36	88.34	50.61
F12	Mean	$1.06\times 10^{9}$	$8.69\times 10^{8}$	$3.55\times 10^{6}$	$2.06\times 10^{7}$	$1.91\times 10^{8}$	$9.73\times 10^{7}$	** $1.35\times 10^{6}$ **
	Std	$3.97\times 10^{8}$	$3.13\times 10^{8}$	$2.26\times 10^{6}$	$2.34\times 10^{7}$	$1.35\times 10^{8}$	$1.47\times 10^{8}$	** $1.12\times 10^{6}$ **
F13	Mean	$6.58\times 10^{8}$	$5.94\times 10^{8}$	$4.10\times 10^{5}$	$1.68\times 10^{5}$	$4.47\times 10^{8}$	$6.18\times 10^{6}$	** $2.05\times 10^{4}$ **
	Std	$3.66\times 10^{8}$	$4.82\times 10^{8}$	$2.60\times 10^{5}$	$8.03\times 10^{4}$	$4.82\times 10^{8}$	$1.79\times 10^{7}$	** $1.97\times 10^{4}$ **
F14	Mean	$1.67\times 10^{6}$	$2.16\times 10^{6}$	$1.03\times 10^{5}$	$1.58\times 10^{5}$	$2.83\times 10^{6}$	** $4.33\times 10^{4}$ **	$4.58\times 10^{5}$
	Std	$6.81\times 10^{5}$	$1.59\times 10^{6}$	$1.04\times 10^{5}$	$2.63\times 10^{5}$	$3.21\times 10^{6}$	** $8.57\times 10^{4}$ **	$3.47\times 10^{5}$
F15	Mean	$2.32\times 10^{7}$	$1.54\times 10^{7}$	$3.21\times 10^{4}$	$9.81\times 10^{4}$	$1.57\times 10^{8}$	$1.53\times 10^{4}$	** 8774.82 **
	Std	$5.65\times 10^{7}$	$2.17\times 10^{7}$	$1.08\times 10^{4}$	$7.14\times 10^{4}$	$2.93\times 10^{8}$	$1.46\times 10^{4}$	** $1.00\times 10^{4}$ **
F16	Mean	2377.04	2357.01	1041.96	1373.83	1513.76	** 756.54 **	1071.52
	Std	336.12	367.99	375.10	345.11	396.41	** 247.68 **	280.82
F17	Mean	1125.15	1197.27	478.85	572.57	793.12	** 325.27 **	672.39
	Std	242.64	315.45	252.01	** 176.78 **	267.21	177.47	260.29
F18	Mean	$4.22\times 10^{6}$	$4.44\times 10^{6}$	** $2.28\times 10^{5}$ **	$1.50\times 10^{6}$	$9.25\times 10^{6}$	$1.01\times 10^{6}$	$1.50\times 10^{6}$
	Std	$6.78\times 10^{6}$	$7.32\times 10^{6}$	** $1.75\times 10^{5}$ **	$2.12\times 10^{6}$	$6.55\times 10^{6}$	$2.87\times 10^{6}$	$1.60\times 10^{6}$
F19	Mean	$1.19\times 10^{8}$	$1.18\times 10^{8}$	$3.51\times 10^{5}$	$5.65\times 10^{5}$	$1.42\times 10^{8}$	$2.84\times 10^{4}$	** $1.30\times 10^{4}$ **
	Std	$1.03\times 10^{8}$	$9.71\times 10^{7}$	$3.17\times 10^{5}$	$6.47\times 10^{5}$	$2.11\times 10^{8}$	$7.13\times 10^{4}$	** $1.37\times 10^{4}$ **
F20	Mean	1074.89	1048.25	649.76	646.39	727.68	** 266.05 **	642.65
	Std	174.04	223.83	178.28	181.80	210.54	** 96.70 **	259.71
F21	Mean	455.79	468.29	289.73	423.16	332.18	** 289.68 **	333.68
	Std	28.65	24.22	** 20.30 **	57.94	24.75	21.12	40.88
F22	Mean	1186.59	1154.18	** 113.27 **	4650.40	3401.53	1683.25	2362.55
	Std	216.88	146.18	** 1.48 **	1624.68	1333.71	1528.96	1711.44
F23	Mean	644.44	633.93	** 473.65 **	630.98	501.06	537.47	535.57
	Std	33.40	27.31	** 26.00 **	85.17	34.26	56.53	59.97
F24	Mean	718.53	710.03	** 521.29 **	683.38	676.19	670.93	615.93
	Std	36.66	25.55	** 23.96 **	71.13	78.01	79.22	57.59
F25	Mean	677.27	654.00	** 385.33 **	474.42	569.89	406.11	394.42
	Std	53.00	46.98	** 1.79 **	44.30	93.20	19.71	15.23
F26	Mean	3771.25	3748.17	1917.15	3625.12	2826.18	** 1828.14 **	2947.50
	Std	581.72	594.17	** 304.23 **	778.68	497.69	691.75	767.10
F27	Mean	685.99	695.75	** 513.55 **	620.23	552.60	590.97	560.40
	Std	65.58	96.51	** 12.04 **	57.54	22.42	65.17	21.40
F28	Mean	906.10	932.23	** 360.75 **	515.52	715.14	602.63	406.18
	Std	** 34.01 **	56.04	46.49	36.52	155.32	334.43	35.27
F29	Mean	2295.67	2191.73	819.40	1525.90	1101.49	** 694.78 **	1106.21
	Std	351.75	271.73	271.88	358.37	264.36	** 118.78 **	299.07
F30	Mean	$2.47\times 10^{8}$	$2.53\times 10^{8}$	$1.40\times 10^{6}$	$2.92\times 10^{6}$	$5.83\times 10^{7}$	$2.78\times 10^{5}$	** 7186.52 **
	Std	$7.86\times 10^{7}$	$1.16\times 10^{8}$	$1.23\times 10^{6}$	$1.55\times 10^{6}$	$7.32\times 10^{7}$	$5.88\times 10^{5}$	** 3772.85 **

**Table 6:** Overall ranking comparison of the compared algorithms on the CEC2017 benchmark functions (best values are shown in bold).

Algorithm	Mean Rank	Best Mean Count	Std Rank	Best Std Count
TLQ-SGWO	**2.20**	9	**2.30**	**13**
RCGA-PSO	2.30	10	3.13	9
PSO	2.33	**11**	3.37	6
WOA	4.27	0	4.70	1
GA	4.57	0	5.07	0
SGWO	6.07	0	4.90	0
GWO	6.27	0	4.53	1

**Table 7:** Pairwise comparison between TLQ-SGWO and the other algorithms on the CEC2017 benchmark functions (best values are shown in bold).

Compared Algorithm	Better Mean Count of TLQ-SGWO	Better Std Count of TLQ-SGWO
GWO	**30/30**	24/30
SGWO	**30/30**	26/30
WOA	27/30	25/30
GA	27/30	28/30
PSO	13/30	20/30
RCGA-PSO	17/30	18/30

**Table 8:** Average Mean-error ranking of the compared algorithms on different types of CEC2017 functions (best values are shown in bold).

Algorithm	F1–F3	F4–F10	F11–F20	F21–F30
TLQ-SGWO	2.67	2.86	2.30	**1.50**
RCGA-PSO	**1.00**	2.00	2.20	3.00
PSO	3.33	**1.71**	**2.10**	2.70
WOA	3.00	5.29	3.40	4.80
GA	5.00	3.71	5.80	3.80
SGWO	6.33	6.14	6.00	6.00
GWO	6.67	6.29	6.20	6.20

**Table 9:** Parameters of cylindrical no-fly zones.

No-Fly Zone	$x_{i}$	$y_{i}$	$z_{i}^{\min}$	$r_{i}$	$h_{i}$	$z_{i}^{\max}$	$\lambda_{i}$
NF1	410	360	223.00	72	225.00	448.00	1.00
NF2	565	545	350.64	58	149.36	500.00	1.10
NF3	535	735	368.43	70	131.57	500.00	1.25
NF4	825	650	178.49	92	260.00	438.49	1.20
NF5	710	435	271.92	66	228.08	500.00	1.15

**Table 10:** Algorithm comparison results in four artificial mountainous scenarios ( best values in each scenario are shown in bold).

**Scenario 1: 2 No-Fly Zones**			**Scenario 2: 3 No-Fly Zones**		
**Algorithm**	**Mean Cost**	**Std**	**Algorithm**	**Mean Cost**	**Std**
GWO	5111.54	1388.68	GWO	6392.25	2327.53
SGWO	4067.56	992.98	SGWO	5589.18	2064.37
TLQ-SGWO	3316.58	** 313.22 **	TLQ-SGWO	** 3728.22 **	** 749.75 **
WOA	7513.29	3104.85	WOA	9366.48	5512.92
GA	6263.89	1517.33	GA	7687.46	1652.77
PSO	4760.03	797.27	PSO	9265.16	2688.02
RCGA-PSO	** 3306.63 **	472.41	RCGA-PSO	6017.83	2344.79
**Scenario 3: 4 No-Fly Zones**			**Scenario 4: 5 No-Fly Zones**		
**Algorithm**	**Mean Cost**	**Std**	**Algorithm**	**Mean Cost**	**Std**
GWO	6807.70	2224.16	GWO	6523.40	1151.39
SGWO	7180.68	1201.16	SGWO	6292.38	1077.95
TLQ-SGWO	** 5542.61 **	** 1051.73 **	TLQ-SGWO	** 5269.77 **	** 610.81 **
WOA	12,089.45	8892.85	WOA	10,932.22	4308.95
GA	8815.55	1674.02	GA	8045.77	1393.97
PSO	10,022.35	3075.20	PSO	9938.52	2411.55
RCGA-PSO	6590.84	1443.35	RCGA-PSO	6004.86	708.20

**Table 11:** Runtime comparison in four artificial mountainous scenarios with the optional TLQ-SGWO safety-redundancy branch disabled (mean ± standard deviation, s).

Algorithm	Scenario 1	Scenario 2	Scenario 3	Scenario 4	Overall
GWO	11.916±0.658	12.434±0.533	15.231±2.926	13.075±1.288	13.164±2.053
SGWO	13.868±0.653	14.583±0.185	14.765±0.760	15.485±0.135	14.675±0.766
TLQ-SGWO	13.020±0.578	12.682±0.402	13.062±0.264	13.130±0.072	12.973±0.407
WOA	12.541±0.655	12.874±0.482	15.325±2.444	13.161±0.416	13.475±1.681
GA	10.767±0.467	11.214±0.402	13.352±2.181	11.586±0.321	11.730±1.490
PSO	16.517±0.847	17.001±0.753	20.979±3.982	17.188±0.562	17.921±2.715
RCGA-PSO	10.895±0.459	11.525±0.420	13.397±2.006	11.755±0.339	11.893±1.394

**Table 12:** Statistical results of the ablation study (20 independent runs).

Ablation Scheme	Mean Cost	Std	Best Cost
A1: Basic SGWO	7272.32	1845.83	4766.52
A2: A1 + initialization/seed paths	6040.75	1006.30	4170.02
A3: A2 + Q-learning strategy scheduling	6784.04	1222.87	4586.91
A4: A3 + Lévy stagnation perturbation	6159.45	1354.51	3885.69
A5: A4 + Alpha terminal refinement	6158.92	1355.41	3883.92
A6: Complete TLQ-SGWO	4877.74	1117.69	3033.46

## Data Availability

Data will be made available on request.

## Abbreviations

The following abbreviations are used in this manuscript:

UAV	Unmanned aerial vehicle
GWO	Grey wolf optimizer
SGWO	Spherical-vector grey wolf optimizer
TLQ	Tent–Logistic hybrid initialization and Q-learning search-strategy scheduling
TLQ-SGWO	Tent–Logistic and Q-learning-based multi-strategy spherical-vector grey wolf optimizer
PSO	Particle swarm optimization
WOA	Whale optimization algorithm
GA	Genetic algorithm
RCGA-PSO	Real-coded genetic algorithm–particle swarm optimization
MaxFEs	Maximum number of function evaluations
Std	Standard deviation
CEC	Congress on Evolutionary Computation
